# A third dose of COVID‐19 mRNA vaccine induces limited humoral response in stem cell transplant recipients who got two vaccine doses before transplant

**DOI:** 10.1002/jha2.637

**Published:** 2022-12-22

**Authors:** Takashi Toya, Daichi Sadato, Takahiro Sanada, Tomoko Honda, Yuya Atsuta, Noritaka Sekiya, Hiroaki Shimizu, Yuho Najima, Takeshi Kobayashi, Yuka Harada, Michinori Kohara, Noriko Doki

**Affiliations:** ^1^ Hematology Division Tokyo Metropolitan Cancer and Infectious Diseases Center, Komagome Hospital Tokyo Japan; ^2^ Clinical Research Support Center Tokyo Metropolitan Cancer and Infectious Diseases Center Komagome Hospital Tokyo Japan; ^3^ Department of Microbiology and Cell Biology Tokyo Metropolitan Institute of Medical Science Tokyo Japan; ^4^ Department of Infection Prevention and Control Tokyo Metropolitan Cancer and Infectious Diseases Center Komagome Hospital Tokyo Japan; ^5^ Department of Clinical Laboratory Tokyo Metropolitan Cancer and Infectious Diseases Center Komagome Hospital Tokyo Japan

**Keywords:** allogeneic hematopoietic stem cell transplantation, COVID‐19, humoral immunity, mRNA vaccine, SARS‐CoV‐2

To the Editor,

Allogeneic hematopoietic stem cell transplantation (HSCT) recipients are at high risk of severe coronavirus disease 19 (COVID‐19) [1, 2], and sufficient times of vaccinations are strongly recommended. In general, post‐HSCT patients should be considered as “never vaccinated” regardless of the pre‐HSCT vaccination history [3]. Limited immunogenicity can be obtained after one dose of severe acute respiratory syndrome coronavirus 2 (SARS‐CoV‐2) vaccine in HSCT recipients [4], and those patients should receive ≥2 vaccine doses several months after transplantation [5, 6]. However, in Japan, many HSCT recipients who had gotten two vaccine doses before transplantation are allowed to get only one mRNA vaccine dose as a “third” dose as of November 2022 due to social constraint, and those patients can be vaccinated “fourth” dose several months later, as “never transplanted”. In this single‐centre study, we prospectively evaluated the humoral immunity before and after one vaccination dose in HSCT recipients who had gotten two vaccination doses before the transplant. This study was performed in accordance with the Declaration of Helsinki and approved by the Ethics Committee of Tokyo Metropolitan Komagome Hospital. Written informed consent was obtained from each patient.

The patients who met all the below criteria were included in this study. (1) adult patients who underwent allogeneic HSCT, (2) patients who received two doses of the COVID‐19 mRNA vaccines before transplant, (3) patients who received one dose of the COVID‐19 mRNA vaccine after HSCT, and (4) patients whose serum samples were collected before and approximately 2–5 weeks after post‐HSCT vaccination. The anti–SARS‐CoV‐2 IgG titer was evaluated in serum samples using an iFlash 3000 chemiluminescence immunoassay analyzer (Shenzhen YHLO Biotech, Shenzhen, China) with an iFlash–SARS‐CoV‐2 IgG kit and iFlash–SARS‐CoV‐2 IgG‐S1 kit, as previously described [7]. The iFlash‐SARS‐CoV‐2 IgG kit primarily detects anti‐nucleocapsid antibodies; thus, patients with positive SARS‐CoV‐2 IgG results were considered to have a previous history of COVID‐19 and were not included in this study. The iFlash–SARS‐CoV‐2 IgG‐S1 kit detects IgG specific to the S1 subunit of the spike (S) protein, which was used to estimate the humoral response of SARS‐CoV‐2 vaccines. According to the manufacturer's instructions, ≥10 arbitrary units (AU)/ml were considered positive. Neutralizing antibody (Nab) levels against SARS‐CoV‐2 were measured using a fully automatic CLIA analyzer (iFlash3000), as previously described [8].

Seven HSCT recipients, who got two vaccine doses median 5 months (range, 4–9) before HSCT, were included. Detailed patient characteristics and transplantation procedures are shown in Table S1. No patient has a history of COVID‐19, which was confirmed by the absence of SARS‐CoV‐2 IgG before and after post‐HSCT vaccination. All donors except the donor in case 4, who received a cord blood transplant, had received at least two COVID‐19 vaccine doses before hematopoietic stem cell donation.

Detailed characteristics of vaccination are shown in Table S2. The median interval from HSCT to post‐HSCT vaccine dose was 7 months (range, 6–8), and administered vaccines before and after transplantation were BNT162b2 in all patients. Five out of seven patients were administered immunosuppressants at vaccination after HSCT.

SARS‐CoV‐2 S1 IgG antibody (Ab) was positive in five of seven patients before post‐HSCT vaccination, although the geometric mean titer (GMT) was as low as 33.41 AU/ml. SARS‐CoV‐2 S1 IgG Ab titer was reevaluated median of 25 days (range, 16–36) after post‐HSCT vaccination, and GMT was 90.27 AU/ml (Figure 1A). Importantly, a marked increase of SARS‐CoV‐2 S1 IgG titer was achieved in only one patient (Case 2), and the median fold change of SARS‐CoV‐2 S1 IgG titer (after/before revaccination) was 1.33 (Figure 1B). We also evaluated the level of Nab against SARS‐CoV‐2 at the same time points. GMT of Nab levels before and after post‐HSCT vaccination was 19.2 and 42.1 AU/ml respectively (Figure 1A). As with SARS‐CoV‐2 S1 IgG, a remarkable increase in Nab level was shown in only one patient (Case 2), and the median fold change of Nab level was 0.986 (Figure 1B). Only case 3 got second post‐HSCT vaccination 3 months after the first post‐HSCT vaccination. SARS‐CoV‐2 S1 IgG and Nab became positive 19 days after the second post‐HSCT vaccination, although the Ab titer was exceedingly low (Figure S1).

**FIGURE 1:**
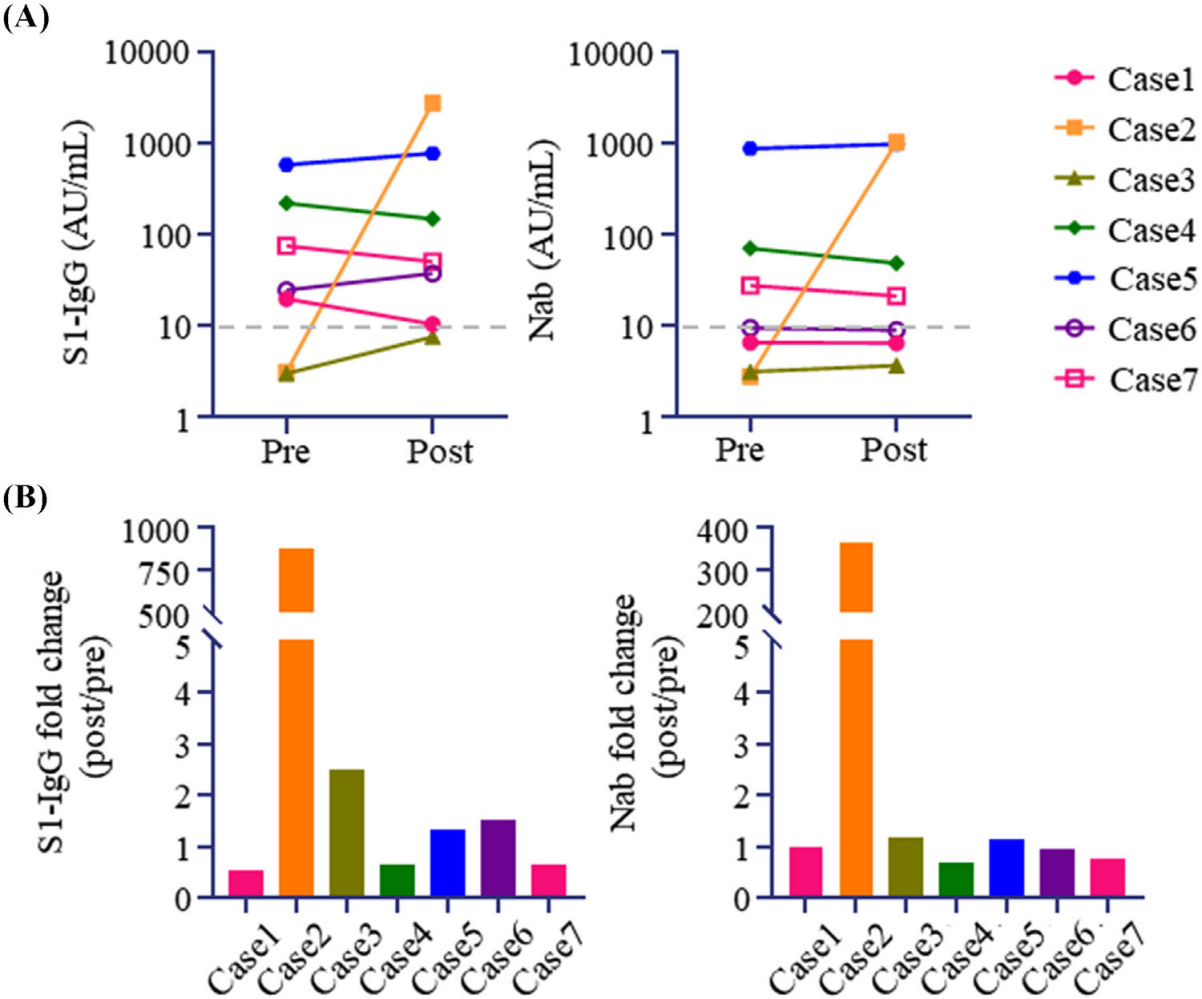
Severe acute respiratory syndrome coronavirus 2 (SARS‐CoV‐2) antibody levels before and after post‐transplantation SARS‐CoV‐2 vaccination. (A) Transition of SARS‐CoV‐2 S1 IgG titer (left) and neutralizing antibody (Nab) level against SARS‐CoV‐2 (right). (B) Fold change of SARS‐CoV‐2 S1 IgG titer (left) and Nab (right) after/before post‐transplant SARS‐CoV‐2 vaccination

In this study, we showed that Ab titer before post‐HSCT vaccination was substantially low in allogeneic HSCT recipients with a history of two vaccine doses before HSCT. In addition, a third vaccine dose 6–8 months after HSCT could induce a limited increase of Ab titer, as opposed to cases with two post‐HSCT vaccination doses [9]. European Conference on Infections in Leukaemia recommends HSCT patients with a previous history of COVID‐19 vaccination before transplant should be vaccinated with a full program [3] and our results validated the appropriateness of the recommendation. In addition, Leclerc et al. have recently reported that the pre‐HSCT vaccination history of the donor rather than the recipient is important for the recipient's humoral response after two post‐HSCT vaccination doses [9]. Considering that humoral response was elicited in only one patient in this study although six of seven donors received mRNA vaccines before hematopoietic stem cell donation, getting vaccines more than one dose promptly seems important for intense humoral response in allogeneic HSCT recipients even though recipients and/or donors have vaccination history. The association between donor Ab levels at donation and humoral immunity in the recipients should be clarified in the future study.

These results should be interpreted with caution because the number of cases in this study is highly limited and patient characteristics are not identical. In our cohort, all patients received a COVID‐19 vaccine dose less than 1 year after HSCT, five out of seven patients suffered from hypogammaglobulinemia, and five patients were administered immunosuppressive therapy, which is common in this population. These characteristics were associated with poor humoral response to the COVID‐19 vaccines among vaccine–naïve HSCT recipients [6, 10] and may also affect the vaccine response in this study.

In conclusion, our preliminary data first suggested that a third dose can induce a limited humoral response in allogeneic HSCT recipients who had gotten two vaccine doses before transplantation. Such individuals should be probably re‐vaccinated as primary vaccine series, that is, ≥2 shots, to enhance the immunogenicity in this highly immunocompromised population.

## AUTHOR CONTRIBUTIONS

Takashi Toya, Daichi Sadato, Noritaka Sekiya, and Michinori Kohara designed the study. Takashi Toya, Yuya Atsuta, Hiroaki Shimizu, Yuho Najima, Takeshi Kobayashi, and Noriko Doki provided medical treatment, gave informed consent, and collected the clinical data. Daichi Sadato and Yuka Harada collected and managed clinical samples. Takahiro Sanada, Tomoko Honda, and Michinori Kohara analyzed the antibody titers. Takashi Toya wrote the manuscript and Daichi Sadato created the figures. Yuka Harada and Noriko Doki supervised this study. All authors have read and approved the manuscript.

## CONFLICT OF INTEREST

The authors declare that they have no conflict of interest.

## FUNDING INFORMATION

The authors received no specific funding for this work.

## Supporting information



Supporting Information

Supporting Information

Supporting Information
