# Supplementary figures and images for: A third dose of COVID‐19 mRNA vaccine induces limited humoral response in stem cell transplant recipients who got two vaccine doses before transplant
Source: EJHaem. 2022 Dec 22;4(1):309–11. doi: 10.1002/jha2.637 (PMC9880636; doi:10.1002/jha2.637)

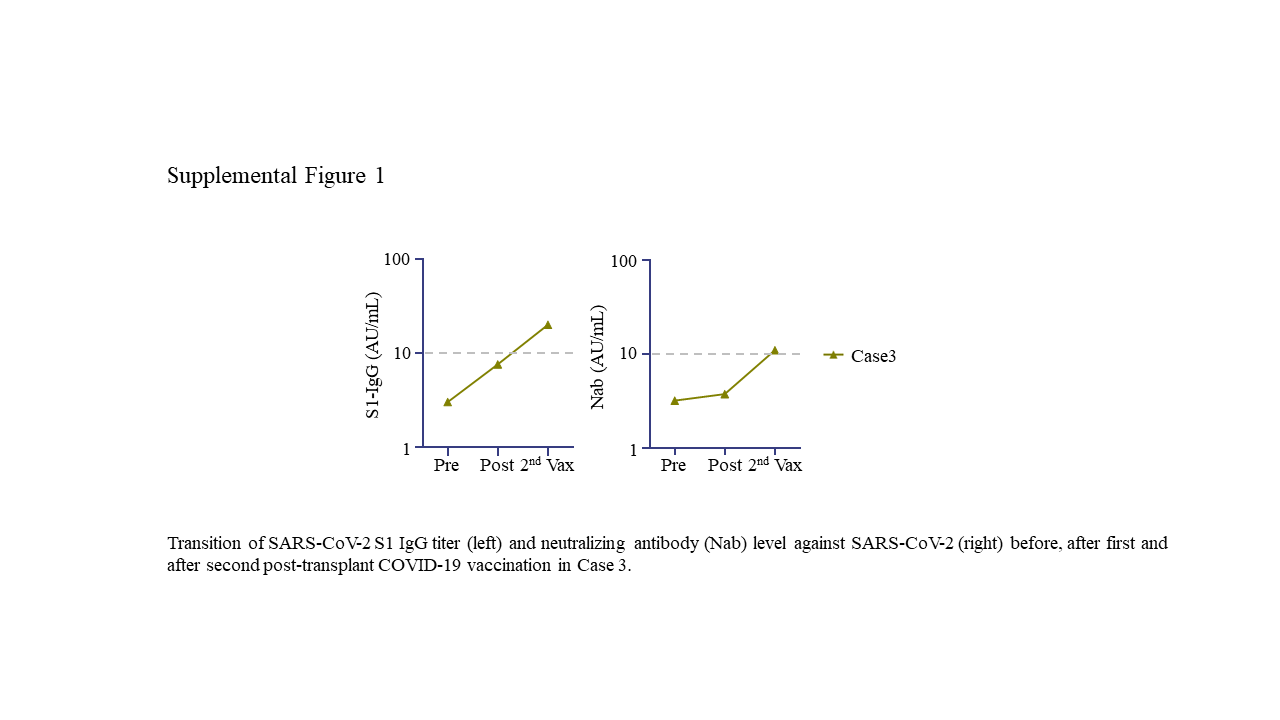

Supplement: Supplementary file 3 — Supporting Information [file JHA2-4-309-s001.tif]
